# Guy's Hospital

**Published:** 1832-04

**Authors:** 


### GUY'S HOSPITAL.

### *Fatal Case of Puncturing a large Thrombus, connected with the Vena Saphæna. Post-mortem Appearances.**

Samuel Basset, æt. forty-seven, a man who had been much in tropical climates and led a dissolute life, presented himself at the surgery on the 26th of July, 1831, having a tumor on the right leg, which he wished to have removed: accordingly, he was admitted into the house under Mr. Key. The tumor was situated immediately below the head of the tibia, a little to the inner side of the leg, in

* Medical Gazette.

the corner of the saphenic vein, was as large as a lemon, somewhat lobulated, and rather softer in some parts than in others. Directly below the tumor was a small, hardened varix: this was evidently connected with it; and the vein, nearly up the whole of the thigh, could be felt hard, like a cord, under the finger. The man stated, that for many years he had had a large vein, in the situation of the tumor, which always disappeared during the night, and he had also been subject to an ulcer of the leg; that about three weeks before his admission, he worked very hard at mowing grass; he then perceived the vein to become larger and inflamed, and for several days it had prevented his working. At the time of his admission, the integuments were livid, but shewed no disposition to ulcerate; the veins in the lower part of the leg were not at all varicose.

A short time after his admission he had an attack of the catarrhal affection, which was so prevalent about that time: by the use of diluents and purgatives, he recovered; and, on August 15th, the tumor was punctured with a lancet, and the contents, consisting of half-coagulated, venous blood, looking like black-currant jelly, squeezed out; no fresh blood was observed to make its escape. The wound was closed by lint, and pressure made with plaster.

16th. A little feverish; slight pain in the leg; rather restless; bowels not open.—A dose of castor oil to be given.

17th. Very restless; thirsty; pain in the head; tongue furred and dry; pulse 120, and irritable; skin hot and dry; leg swelled, a little inflamed; constant nausea, with occasional vomiting; tumor rather painful, plaster removed; a small quantity of thin sanious matter, a little half-decomposed blood, and also a very small portion of fresh escaped.—A poultice to be applied to the tumor; evaporating lotion to the leg. ℞. Tinct. Hyoscyam. ♏xx. c. Mist. Effervescent. 6ta horâ sumend.

18th. Exceedingly restless, constantly tossing about, yet is stupid and rather delirious; constant vomiting of green, bily mucus; tongue red and cracked; skin dry; leg more swelled, the integuments inflamed and hot; does not complain of more pain in the course of the vein than elsewhere; bowels opened; pulse 125, weak.—℞. Ammon. Subcarb. gr. xx. Infus. Serpentariæ ℥iss. in statu effervescentiæ c. succo limonis 6ta hora.

19th, A.M. No sleep; delirious; constantly muttering; tongue dry, cracked, and brown; sickness not so constant; pain on pressure over the liver; leg not quite so much inflamed; thigh a little so, as is the opposite foot, which, he says, is painful; pulse 130, small, and soft. The tumor laid open to its whole length, and a piece of lint introduced; the surface of it hard, dry, and irregular. —Continue the poultice and cold wash. ℞. Liq. Ammon. Acet. ℥ss., Tinct. Hyos. ♏xxx. ex Aquæ Menthæ, 6ta hora. Mustard poultice to the pit of the stomach.

P.M. No better; delirious; pulse small and very rapid; tongue brown; sordes on teeth; excessively thirsty; sickness stopped.

—℞. Hydr. Submuriatis gr. ij. Opii gr. ij. hora somni sumend., and some brandy and soda-water occasionally.

20th, A.M. No sleep; constantly muttering; bowels opened; does not answer when spoken to; no sickness; pulse rapid and feeble; a little subsultus tendinum.—Continue brandy with the soda-water.

P.M. Fast sinking; subsultus increased; pulse scarcely perceptible; teeth covered with sordes; leg of much the same appearance; is quite insensible.

21st. Died at ten this morning: a few hours before death, there was perceived to be a little swelling, as though matter were forming immediately about the right wrist; if this were pressed upon, he shewed some signs of feeling, and to this only was he sensible.

*Post-mortem appearances, twenty-seven hours after death.* Body in a very putrid state: vena saphena inflamed from the tumor to within two inches of the place where it joins the femoral vein, at which part there was a natural coagulum: below this, to the varix, the coats were much thickened, and containing a sanguino-purulent fluid adhering tenaciously to the vein. The tumor was a dilated part of the vein itself; the coats of it were exceedingly thickened and irregular. The communication between the tumor and veins in the lower part of the leg was effectually prevented by the obliterated small varix, mentioned as being felt attached to the larger one; and the vein above was so thickened and contracted as only to allow of a probe being passed from the vein above to the sac below.

Into one only of the veins of the leg had the inflammation apparently extended: this contained a small quantity of healthy pus; all the other branches were healthy, and contained coagula; vena cava, femoral, and iliac veins natural; heart large and empty; lungs gorged with blood; spleen larger than natural; kidneys soft and mottled.

In the right arm, directly above the wrist, the cellular membrane was observed to be inflamed, and some serum to have been thrown out: this, probably, had the patient lived a little longer, would have gone on to suppuration, deposits of pus being one of the characteristic signs of phlebitis.

---

